# Gliotoxin, a Known Virulence Factor in the Major Human Pathogen Aspergillus fumigatus, Is Also Biosynthesized by Its Nonpathogenic Relative *Aspergillus fischeri*

**DOI:** 10.1128/mBio.03361-19

**Published:** 2020-02-11

**Authors:** Sonja L. Knowles, Matthew E. Mead, Lilian Pereira Silva, Huzefa A. Raja, Jacob L. Steenwyk, Gustavo H. Goldman, Nicholas H. Oberlies, Antonis Rokas

**Affiliations:** a Department of Chemistry and Biochemistry, University of North Carolina at Greensboro, Greensboro, North Carolina, USA; b Department of Biological Sciences, Vanderbilt University, Nashville, Tennessee, USA; c Faculdade de Ciencias Farmacêuticas de Ribeirão Preto, Universidade de São Paulo, São Paulo, Brazil; Yonsei University; Maynooth University, Ireland; University of Wisconsin—Madison

**Keywords:** fungal pathogenesis, secondary metabolism, gliotoxin, specialized metabolism, evolution of virulence, *laeA*, aspergillosis

## Abstract

Aspergillus fumigatus is a major opportunistic human pathogen. Multiple traits contribute to A. fumigatus pathogenicity, including its ability to produce specific secondary metabolites, such as gliotoxin. Gliotoxin is known to inhibit the host immune response, and genetic mutants that inactivate gliotoxin biosynthesis (or secondary metabolism in general) attenuate A. fumigatus virulence. The genome of *Aspergillus fischeri*, a very close nonpathogenic relative of A. fumigatus, contains a biosynthetic gene cluster that is homologous to the A. fumigatus gliotoxin cluster. However, *A. fischeri* is not known to produce gliotoxin. To gain further insight into the similarities and differences between the major pathogen A. fumigatus and the nonpathogen *A. fischeri*, we examined whether *A. fischeri* strain NRRL 181 biosynthesizes gliotoxin and whether the production of secondary metabolites influences the virulence profile of *A. fischeri*. We found that *A. fischeri* biosynthesizes gliotoxin under the same conditions as A. fumigatus. However, whereas loss of *laeA*, a master regulator of secondary metabolite production (including gliotoxin biosynthesis), has previously been shown to reduce A. fumigatus virulence, we found that *laeA* loss (and loss of secondary metabolite production) in *A. fischeri* does not influence its virulence. These results suggest that LaeA-regulated secondary metabolites are virulence factors in the genomic and phenotypic background of the major pathogen A. fumigatus but are much less important in the background of the nonpathogen *A. fischeri*. Understanding the observed spectrum of pathogenicity across closely related pathogenic and nonpathogenic *Aspergillus* species will require detailed characterization of their biological, chemical, and genomic similarities and differences.

## Abstract (precis)

Aspergillus fumigatus is a major opportunistic fungal pathogen of humans, but most of its close relatives are nonpathogenic. Why is that so? This important, yet largely unanswered, question can be addressed by examining how A. fumigatus and its close nonpathogenic relatives are similar or different with respect to virulence-associated traits. We investigated whether Aspergillus fischeri, a nonpathogenic close relative of A. fumigatus, can produce gliotoxin, a mycotoxin known to contribute to A. fumigatus virulence. We discovered that the nonpathogenic A. fischeri produces gliotoxin under the same conditions as those of the major pathogen A. fumigatus. However, we also discovered that, in contrast to what has previously been observed in A. fumigatus, the loss of secondary metabolite production in *A. fischeri* does not alter its virulence. Our results are consistent with the “cards of virulence” model of opportunistic fungal disease, in which the ability to cause disease stems from the combination (“hand”) of virulence factors (“cards”) but not from individual factors *per se*.

## OBSERVATION

**FIG 1:**
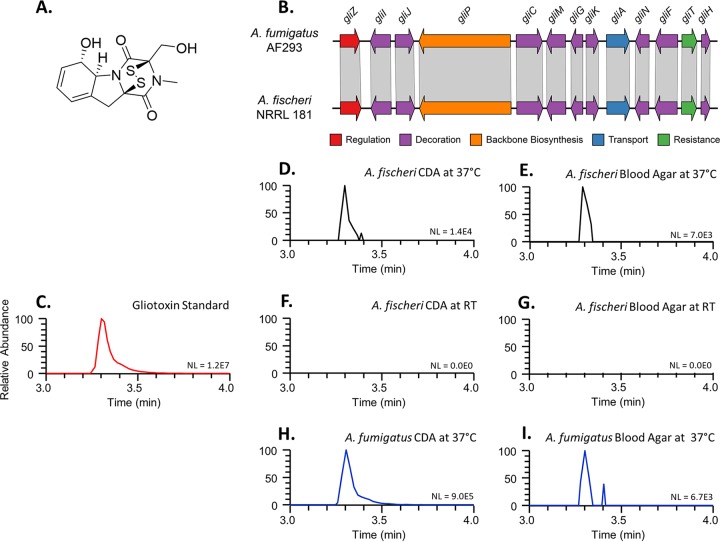
*Aspergillus fischeri* biosynthesizes gliotoxin when grown under conditions that induce A. fumigatus gliotoxin biosynthesis. (A) Chemical structure of gliotoxin. (B) The genome of the nonpathogenic species *A. fischeri* strain NRRL 181 (14, 28) contains a biosynthetic gene cluster homologous to the gliotoxin cluster in the major pathogen A. fumigatus strain Af293 (4–6). Arrows indicate genes and the direction in which they are transcribed. Homologous genes are connected by gray parallelograms. (C to I) Chromatograms demonstrating the biosynthesis of gliotoxin in *A. fischeri* when grown on CDA or blood agar at 37°C. Each sample (dried organic extract in MeOH at a concentration of 0.2 mg/ml) was analyzed by UHPLC-HRESIMS, and the data are presented as extracted ion chromatograms (XIC) using the protonated mass of gliotoxin (C_13_H_15_N_2_O_4_S_2_; [M+H]^+^ = 327.0473) and a window of ± 5.0 ppm. (C) Analysis of the gliotoxin standard (in MeOH at a concentration of 0.01 mg/ml). (D) A. fumigatus grown on CDA at 37°C. (E) A. fumigatus grown on blood agar at 37°C. (F) *A. fischeri* grown on CDA incubated at 37°C. (G) *A. fischeri* grown on blood agar incubated at 37°C. (H) *A. fischeri* grown on CDA at room temperature (RT). (I) *A. fischeri* grown on blood agar at RT. The retention time (3.30 min) and accurate mass (327.0473 ± 5.0 ppm) data confirm the biosynthesis of gliotoxin by *A. fischeri* in panels F and G. NL, normalization level (i.e., base peak intensity; the larger the NL value the better the signal to noise ratio).

Aspergillus fumigatus is a major fungal pathogen responsible for hundreds of thousands of infections and deaths each year (1, 2). Several secondary metabolites biosynthesized by A. fumigatus have been shown to be required for disease (3). For example, gliotoxin (Fig. 1A), a secondary metabolite that belongs to the epipolythiodioxopiperazine (ETP) class of mycotoxins (4–6), can be detected in the sera of patients with invasive aspergillosis (7) and is known to inhibit the host immune response (3). When the *gliP* gene, which encodes the essential nonribosomal peptide synthetase of the gliotoxin biosynthetic gene cluster, is deleted from A. fumigatus, the mutant strain does not biosynthesize gliotoxin and exhibits attenuated virulence in a nonneutropenic murine model of aspergillosis (8–10). Similarly, deletion of *laeA*, a positive regulator of several A. fumigatus secondary metabolites, including gliotoxin, also reduces virulence (11, 12). These results suggest that gliotoxin, as well as other secondary metabolites, contributes to A. fumigatus virulence (3).

Even though A. fumigatus is a major pathogen, its closest relatives are nonpathogenic (13–15). For example, the closely related species *Aspergillus fischeri* has been identified as the cause of only a few clinical cases (16, 17) and is not considered pathogenic. Detailed comparisons of levels of virulence in diverse murine and invertebrate models of fungal disease have shown that *A. fischeri* is much less virulent than A. fumigatus (14). It is important to emphasize here that nonpathogens can sometimes cause disease in diverse animal models of aspergillosis, especially when high inoculums of spores are administered, as we have observed in previous experiments with *A. fischeri*; however, in all such cases, nonpathogens exhibit lower levels of virulence than pathogens (14).

Despite significant differences in their abilities to cause disease, a recent examination of known genetic contributors to virulence revealed that nearly all genes known to contribute to A. fumigatus disease are also present in *A. fischeri* (14). For example, both species appear to contain *laeA*, and deletion of the *laeA* gene from either species reduces the biosynthesis of secondary metabolites (14, 18), suggesting that the gene’s function is conserved. Similarly, both species appear to contain intact gliotoxin biosynthetic gene clusters (Fig. 1B); however, while gliotoxin production has been shown in A. fumigatus and a few other closely related species (19), it has not been reported to be produced by *A. fischeri* (14, 19). These data raise two questions: is *A. fischeri* capable of biosynthesizing gliotoxin, and if it is, how does the production of gliotoxin, and secondary metabolites more generally, influence its virulence profile?

## 

### *A. fischeri*, a nonpathogenic relative of the major pathogen A. fumigatus, can also biosynthesize gliotoxin.

To test whether *A. fischeri* biosynthesizes gliotoxin, we examined the chemical profile of a standard of gliotoxin, an extract of A. fumigatus strain Af293, and an extract of *A. fischeri* strain NRRL 181 via ultrahigh-performance liquid chromatography–high-resolution electrospray ionization mass spectrometry (UHPLC-HRESIMS). We collected three sets of data: chromatographic retention time, high-resolution mass spectrometry data, and tandem mass spectrometry fragmentation patterns. Analysis of a gliotoxin standard (Fig. 1C) showed that it elutes at 3.30 min with an accurate mass of 327.0464 Da (2.8 ppm) and has key fragments of 263.1 Da and 245.1 Da, in accord with values reported in the literature (20).

We next analyzed A. fumigatus strain Af293 as a positive control since it is known to biosynthesize gliotoxin (19). When A. fumigatus was grown on Czapek-Dox agar (CDA) at 37°C (Fig. 1D), a peak with the same retention time (3.30 min), HRESIMS spectrum, MS/MS spectrum, and accurate mass of 327.0463 (3.1 ppm) as the gliotoxin standard was noted (Fig. S1 and S2). We also detected gliotoxin production, albeit in lower abundance, when we cultured A. fumigatus on 5% blood agar at 37°C (Fig. 1E). In contrast, we did not observe gliotoxin production when we grew A. fumigatus on oatmeal agar (OMA) at 37°C (Fig. S3).

10.1128/mBio.03361-19.1 FIG S1 The mass spectra of gliotoxin in A. fumigatus grown on CDA and blood agar at 37°C verify the biosynthesis of gliotoxin by cultures of A. fumigatus on both CDA and blood agar at 37°C. Data are presented as mass-to-charge ratios (*m/z*). Download FIG S1, DOCX file, 0.4 MB. Copyright © 2020 Knowles et al. 2020 Knowles et al. This content is distributed under the terms of the Creative Commons Attribution 4.0 International license.

10.1128/mBio.03361-19.2 FIG S2 The fragmentation pattern (i.e., MS/MS data) of gliotoxin in A. fumigatus grown on CDA and blood agar at 37°C verify the biosynthesis of gliotoxin in both the CDA and blood agar growths of A. fumigatus at 37°C. Download FIG S2, DOCX file, 0.3 MB. Copyright © 2020 Knowles et al. 2020 Knowles et al. This content is distributed under the terms of the Creative Commons Attribution 4.0 International license.

10.1128/mBio.03361-19.3 FIG S3 Base peak chromatograms of the gliotoxin standard, A. fumigatus grown on OMA at 37°C, *A. fischeri* grown on OMA at 37°C, *ΔlaeA A. fischeri* grown on CDA at 37°C, and *ΔlaeA A. fischeri* grown on blood agar at 37°C show that some media do not induce gliotoxin biosynthesis. Download FIG S3, DOCX file, 0.2 MB. Copyright © 2020 Knowles et al. 2020 Knowles et al. This content is distributed under the terms of the Creative Commons Attribution 4.0 International license.

To test whether *A. fischeri* biosynthesized gliotoxin, we grew strain NRRL 181 on the same media and under the same temperature conditions as A. fumigatus. When *A. fischeri* was grown on CDA at 37°C, we observed a peak with the same retention time (Fig. 1F), HRESIMS spectrum (Fig. S4), and MS/MS spectrum as those observed when analyzing our A. fumigatus extract (Fig. S5), indicating gliotoxin biosynthesis in *A. fischeri*. Similarly, we detected gliotoxin production in lower abundance when we grew *A. fischeri* on 5% blood agar at 37°C (Fig. 1G). In contrast, we did not observe gliotoxin production when we grew *A. fischeri* on CDA or on 5% blood agar at room temperature (Fig. 1H or I, respectively) or on oatmeal agar at 37°C (Fig. S3). These results demonstrate that (ii) the nonpathogen *A. fischeri* biosynthesizes similar quantities of gliotoxin under the same conditions that induce gliotoxin biosynthesis in the major pathogen A. fumigatus and (ii) as with what has previously been observed in A. fumigatus (21, 22), both growth medium and temperature influence gliotoxin biosynthesis in *A. fischeri*.

10.1128/mBio.03361-19.4 FIG S4 The mass spectra of gliotoxin in *A. fischeri* verify the biosynthesis of gliotoxin by cultures of *A. fischeri* on both CDA and blood agar at 37°C. Download FIG S4, DOCX file, 0.3 MB. Copyright © 2020 Knowles et al. 2020 Knowles et al. This content is distributed under the terms of the Creative Commons Attribution 4.0 International license.

10.1128/mBio.03361-19.5 FIG S5 The fragmentation patterns (i.e., MS/MS data) of gliotoxin in *A. fischeri* further verify the biosynthesis of gliotoxin by cultures of *A. fischeri* in both CDA and blood agar at 37°C. Download FIG S5, DOCX file, 0.3 MB. Copyright © 2020 Knowles et al. 2020 Knowles et al. This content is distributed under the terms of the Creative Commons Attribution 4.0 International license.

### *laeA*, a master regulator of secondary metabolism and A. fumigatus virulence factor, is not a virulence factor in *A. fischeri.*

**FIG 2:**
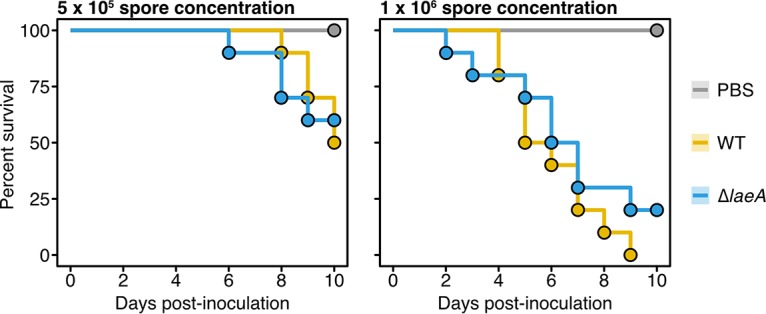
Deletion of the master regulator *laeA* in *A. fischeri* does not alter its virulence. Cumulative survivals of moth (Galleria mellonella) larvae inoculated with 5 × 10^5^ (left) or 1 × 10^6^ (right) asexual spores or conidia of either the Δ*laeA* mutant or the wild-type (WT) *A. fischeri* NRRL 181 strain are shown. Comparisons of moth cumulative survival when infected with either the Δ*laeA* or WT strain revealed no statistically significant differences at spore concentrations of 5 × 10^5^ (left) or 1 × 10^6^ (right) (*P* value = 0.91 and 0.30, respectively; log-rank test). For the inoculations, 10 moths were infected per group.

To test whether the regulation of secondary metabolite production contributes to the virulence profile of *A. fischeri*, we deleted the endogenous copy of *laeA* from *A. fischeri* and infected larvae of the moth Galleria mellonella, a well-established invertebrate model of fungal disease (23), with the resulting mutant strain (unpublished data). The use of G. mellonella larvae is an appropriate model for our study for two reasons. First, our previous work revealed consistent virulence profile differences between wild-type (WT) strains of A*. fischeri* and A. fumigatus in two different murine models and in G. mellonella moth larvae (14). Second, infection of G. mellonella larvae with A. fumigatus is known to induce gliotoxin biosynthesis (24). We infected asexual spores (conidia) at two different concentrations and compared the survival curves between the Δ*laeA* mutant and the WT strain of *A. fischeri* (Fig. 2). At both concentrations, our experiments showed that levels of moth larval survival were not significantly different between the Δ*laeA* and WT strains.

Importantly, the Δ*laeA* strain of *A. fischeri* NRRL 181 is known to exhibit reduced production of secondary metabolites under diverse conditions in a manner consistent with the gene’s role as a master regulator of secondary metabolism (14). To confirm that the Δ*laeA* strain does not produce gliotoxin, we analyzed it using the same chemical methods that showed the production of gliotoxin in the WT strain following growth on CDA or 5% blood agar at 37°C. Unlike with the WT strain (Fig. 1F and G), we did not observe gliotoxin production in the Δ*laeA* strain (Fig. S3). Although the losses of *laeA* and secondary metabolite, including gliotoxin, production have previously been shown to reduce the virulence of the major pathogen A. fumigatus (11, 12), our results suggest that the losses of *laeA* and secondary metabolite production (14) in *A. fischeri* do not influence its virulence.

Deletion of *laeA* (11, 12) and *gliP* (8–10) results in the attenuation of A. fumigatus virulence; in contrast, deletion or overexpression of *gliZ*, the transcriptional regulator of the gliotoxin biosynthetic cluster, does not alter the virulence of A. fumigatus (25). Dissecting the effect of gliotoxin in *A. fischeri* virulence through the construction of Δ*gliZ* and Δ*gliP* mutants in multiple animal models would be an interesting follow-up experiment, especially given that A. fumigatus Δ*laeA* strains have previously been shown to produce a lower, but considerable, amount of gliotoxin *in vivo* during murine infection (25). However, given that the deletion of *laeA* does not alter *A. fischeri* virulence (Fig. 2), the expectation is that specific inactivation of the gliotoxin biosynthetic gene cluster would not alter the virulence profile of *A. fischeri*.

### Concluding remarks.

In this study, we show for the first time that *A. fischeri*, when grown under conditions known to induce gliotoxin production in A. fumigatus, can biosynthesize gliotoxin (Fig. 1). Furthermore, we show that an *A. fischeri* mutant that lacks a master regulatory gene of secondary metabolism (*laeA*) does not alter the pathogenic potential of *A. fischeri* (Fig. 2). Thus, it appears that secondary metabolites are virulence factors in the genomic and phenotypic background of the pathogen A. fumigatus but that they are much less important for virulence in the genomic background of the nonpathogen *A. fischeri*. These results provide support for the “cards of virulence” model of opportunistic fungal disease (26), in which the ability to cause disease stems from the combination (“hand”) of individual virulence factors (“cards”). We hypothesize that while *A. fischeri* possesses the cards for gliotoxin production and secondary metabolism regulation, its cumulative hand is thankfully not a winner when it comes to causing disease.

### Fungal strains.

*Aspergillus fischeri* strain NRRL 181 was obtained from the ARS Culture Collection (NRRL) (14). A. fumigatus strain Af293 was also utilized as a positive control (27).

### Growth conditions.

All strains were maintained on potato dextrose agar (PDA; Difco). To establish individual cultures, an agar square along with fungal mycelium was cut out aseptically from the leading edge of the culture and transferred onto blood agar (tryptic soy agar with 5% sheep’s blood; Hardy Diagnostics), Czapek-Dox agar (CDA; Difco), or oatmeal agar (OMA; Difco). All cultures at 37°C were maintained in an incubator (VWR International) in the dark for 4 days. All cultures at room temperature (RT; ∼22°C) were kept for 2 weeks under a 12-h light/12-h dark cycle. *A. fischeri* was grown on CDA (RT and 37°C), blood agar (RT and 37°C), and OMA (37°C). A. fumigatus was grown on CDA (37°C), blood agar (37°C), and OMA (37°C).

### Extraction.

To evaluate the biosynthesis of gliotoxin in these fungal strains, cultures were extracted with organic solvents and analyzed by mass spectrometry (see below). The fungus was extracted from agar plates by spraying the fungal mycelium with methanol (MeOH), chopping it with a spatula, and transferring the contents to a scintillation vial. Acetone (∼15 ml) was then added to the scintillation vial, and the resulting slurry was vortexed vigorously for approximately 3 min before being steeped for 4 h at RT. Subsequently, the mixture was filtered, and the resulting material was dried under a stream of nitrogen gas to yield the dried organic extract. Solid medium was prepared and extracted as reported previously (14).

### UHPLC-HRESIMS analysis.

High-resolution electrospray ionization mass spectrometry (HRESIMS) experiments utilized a Thermo LTQ Orbitrap XL mass spectrometer (Thermo Fisher Scientific), equipped with an electrospray ionization source. This was coupled to an Acquity ultrahigh-performance liquid chromatography (UHPLC) system (Waters Corp.), using a flow rate of 0.3 ml/min and a bridged ethylene hybrid C_18_ column (2.1 mm by 50 mm, 1.7 μm) that was operated at 40°C. The mobile phase consisted of CH_3_CN-H_2_O (Fischer Optima LC-MS grade; both were acidified with 0.1% formic acid). The gradient began at 15% CH_3_CN and increased linearly to 100% CH_3_CN over 8 min, at which point it was held for 1.5 min before it was returned to starting conditions to reequilibrate.

Extracts were analyzed in the positive-ion mode, with scanning over a mass range of *m/z* 100 to 2,000 at a resolving power of 30,000. The spray voltage, source capillary, and tube lens voltages were set to 4.0 kV, 20 V, and 100 V, respectively, with a nitrogen sheath gas set to 30 arbitrary units (arb) and a capillary temperature at 300°C. The fragmentation patterns (i.e., MS/MS data) were obtained by using an inclusion list containing the mass of gliotoxin ([M+H]^+^ = 327.047 *m/z*), with an isolation window of 2 Da and a collision energy of 35%. The dried organic extracts and gliotoxin standard (Cayman Chemical Company) were prepared in MeOH at a concentration of 0.2 mg/ml and 0.01 mg/ml, respectively, with an injection volume of 3 μl. To eliminate the possibility for sample carryover, two blanks (MeOH) were injected between every sample injection, and the gliotoxin standard was analyzed at the end of the run.

### Virulence studies using an invertebrate model of fungal disease (Galleria mellonella).

Virulence experiments were performed as previously described (14). Briefly, larvae of the moth G. mellonella were obtained by breeding adult moths (23) and selecting larvae that were similar in size (∼275 to 330 mg). Prior to use, all larvae were kept for 24 h in glass petri dishes in darkness at 37°C. Asexual spores (conidia) of Δ*laeA* mutant or the wild-type (WT) *A. fischeri* strains were obtained by growing the organism on a yeast extract-agar-glucose (YAG) medium for 2 days. Conidia were harvested in phosphate-buffered saline (PBS) and filtered through Miracloth (Calbiochem). Conidial concentration was estimated using a hemocytometer, and conidial viability was assessed through incubation on YAG medium at 37°C for 48 h.

For infection assays, 10 G. mellonella larvae in the final (sixth) instar larval stage of development were used per condition. Each larva in the test group was infected with a 5-μl inoculum of conidia from the Δ*laeA* mutant of *A. fischeri* (at a concentration of either 5 × 10^5^ spores/μl or 1 × 10^6^ spores/μl), whereas each larva in the control group was inoculated with the same concentration of the WT strain of *A. fischeri*. All inoculations were done using a Hamilton syringe (7000.5KH). All injections were performed at the hemocoel of each larva via the last left proleg. Following inoculation, all larvae were incubated in glass petri dishes in darkness at 37°C. Larval killing was scored daily. Larvae were considered dead if they did not move in response to touch.
