# Supplementary figures and images for: Gliotoxin, a Known Virulence Factor in the Major Human Pathogen Aspergillus fumigatus, Is Also Biosynthesized by Its Nonpathogenic Relative Aspergillus fischeri
Source: mBio. 2020 Feb 11;11(1):e03361-19. doi: 10.1128/mBio.03361-19 (PMC7018655; doi:10.1128/mBio.03361-19)

**FIG S1**


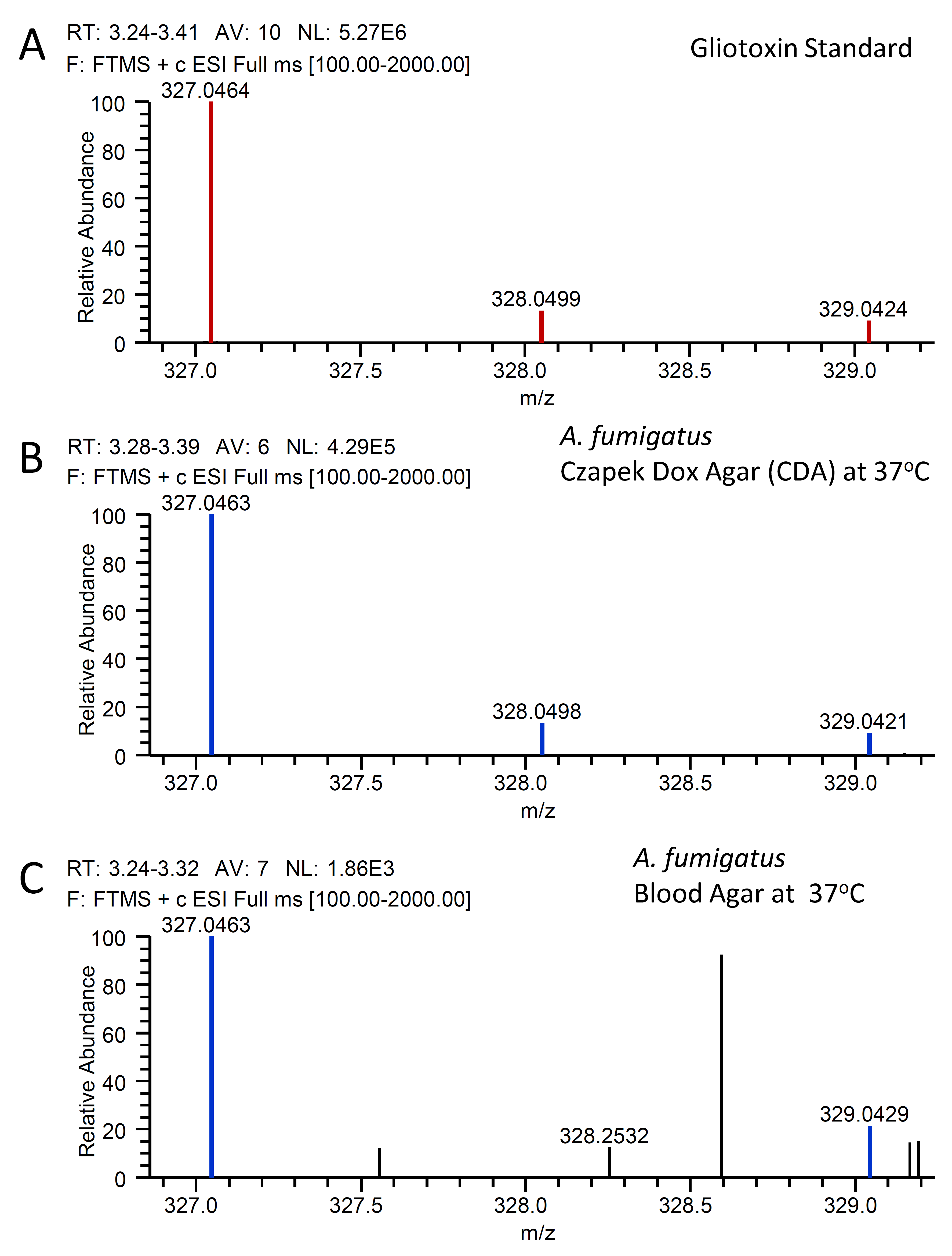

Supplement: FIG S1 [file mBio.03361-19-sf001.docx]

**FIG S2**


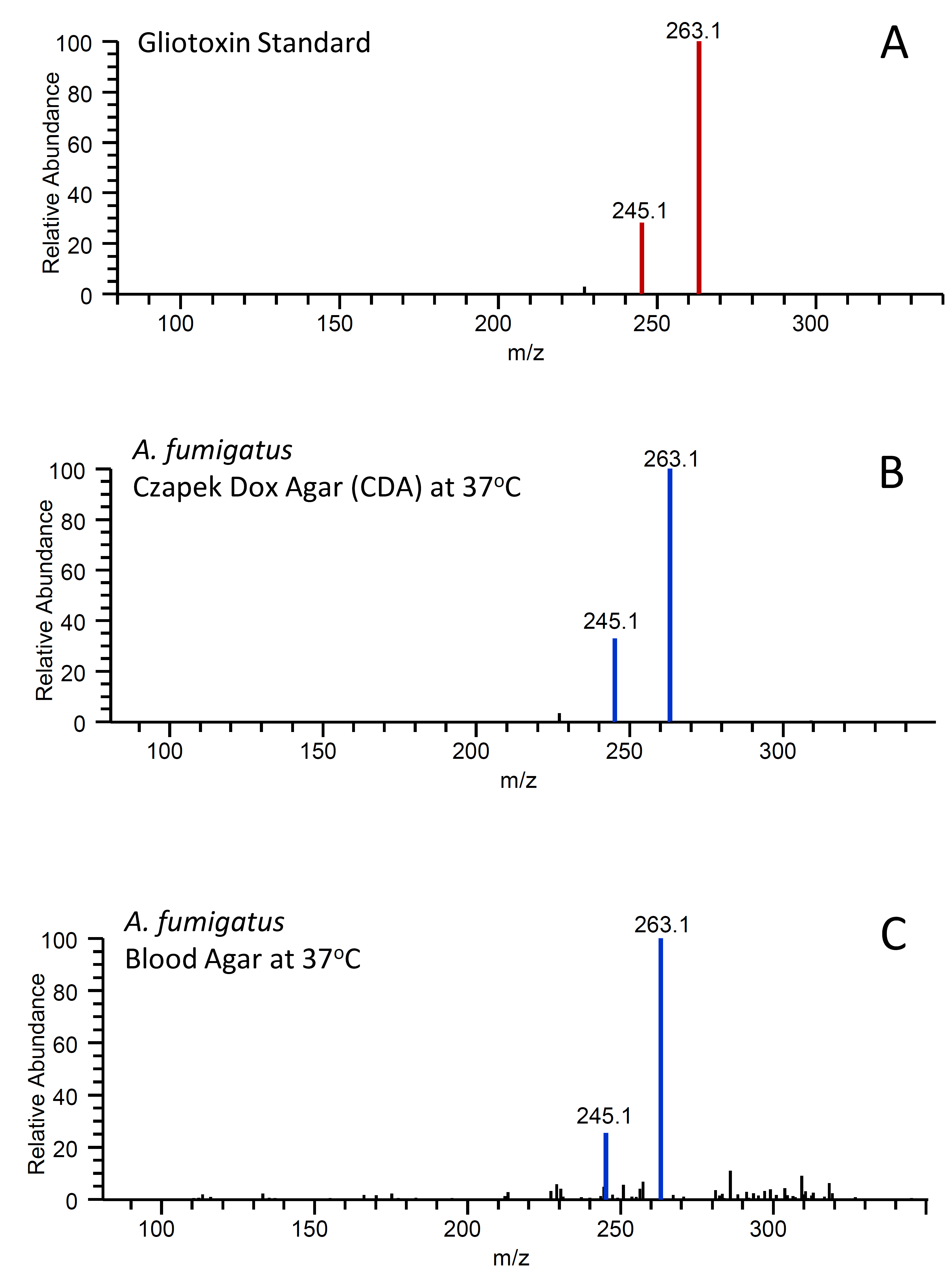

Supplement: FIG S2 [file mBio.03361-19-sf002.docx]

**FIG S3**


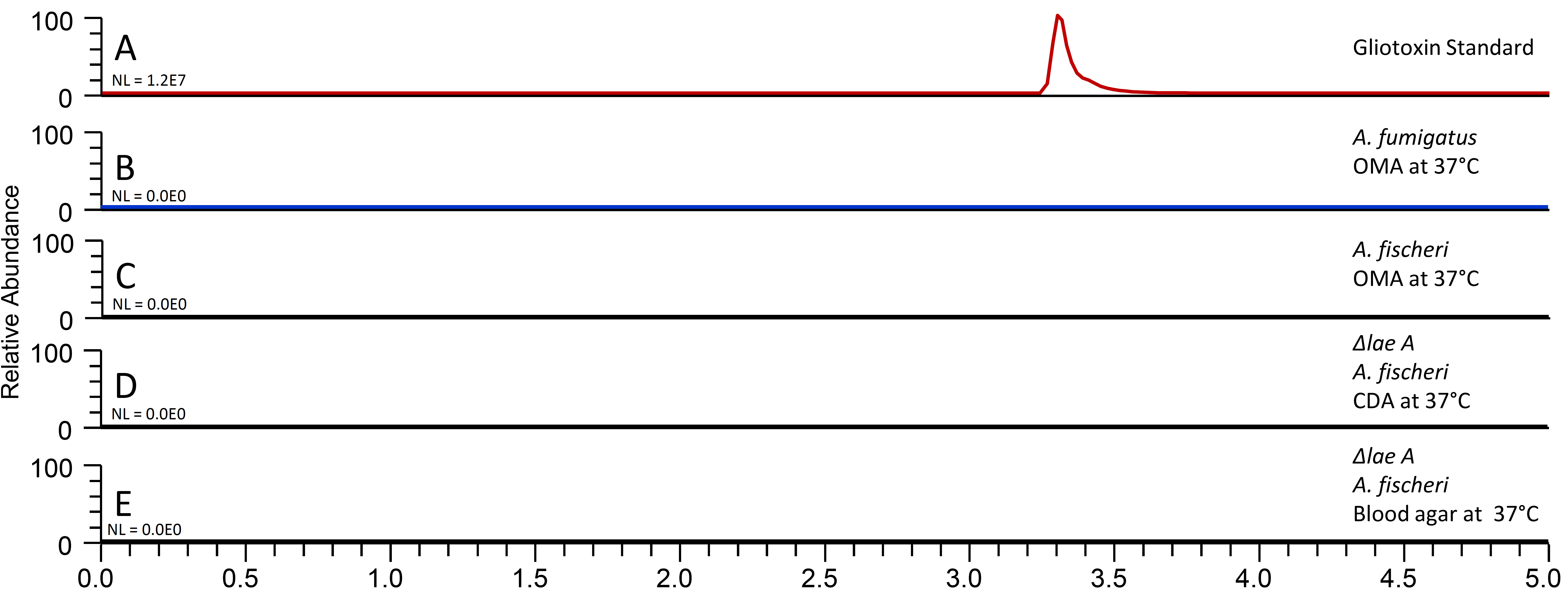

Supplement: FIG S3 [file mBio.03361-19-sf003.docx]

**FIG S4**


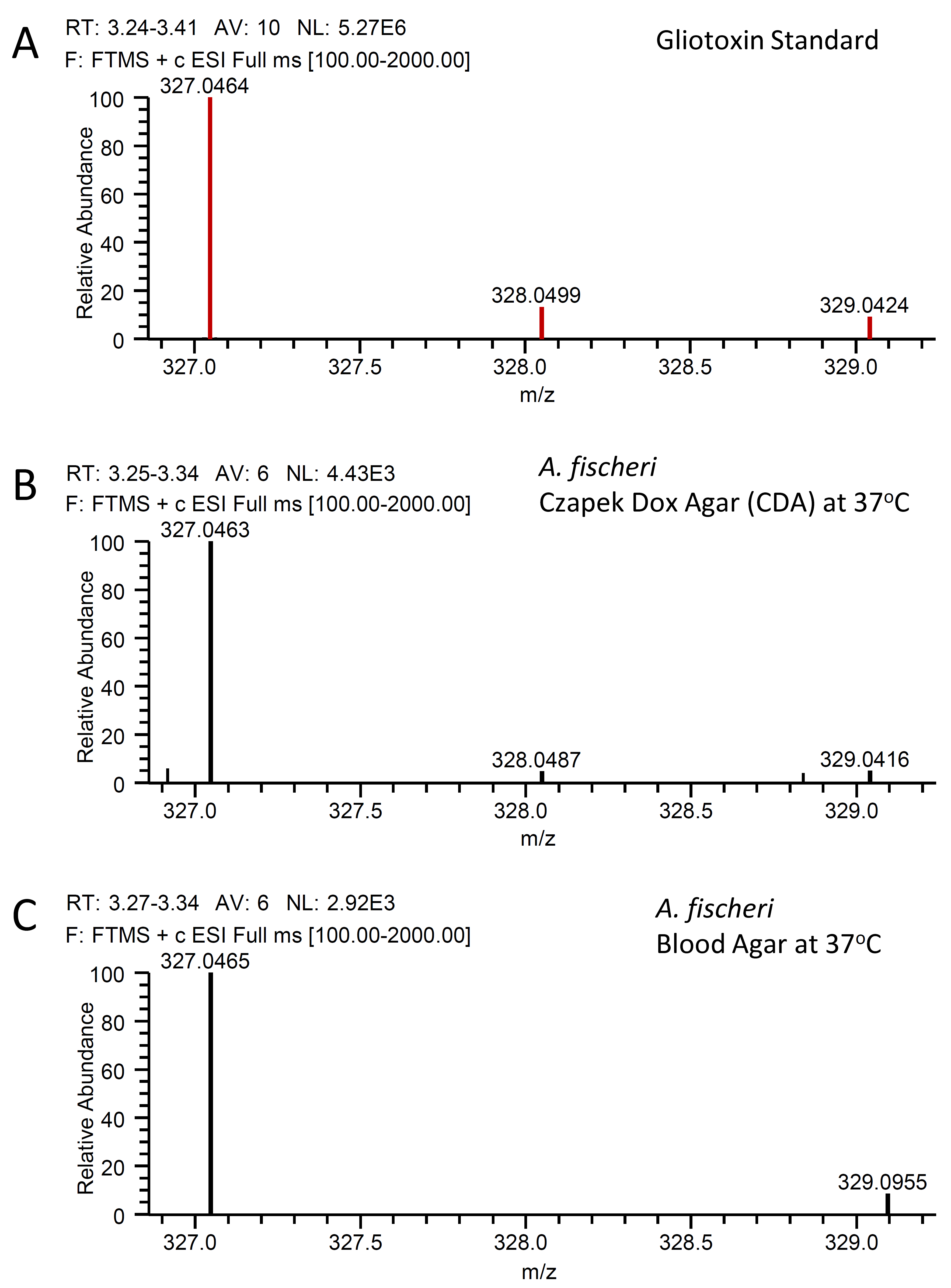

Supplement: FIG S4 [file mBio.03361-19-sf004.docx]

**FIG S5**


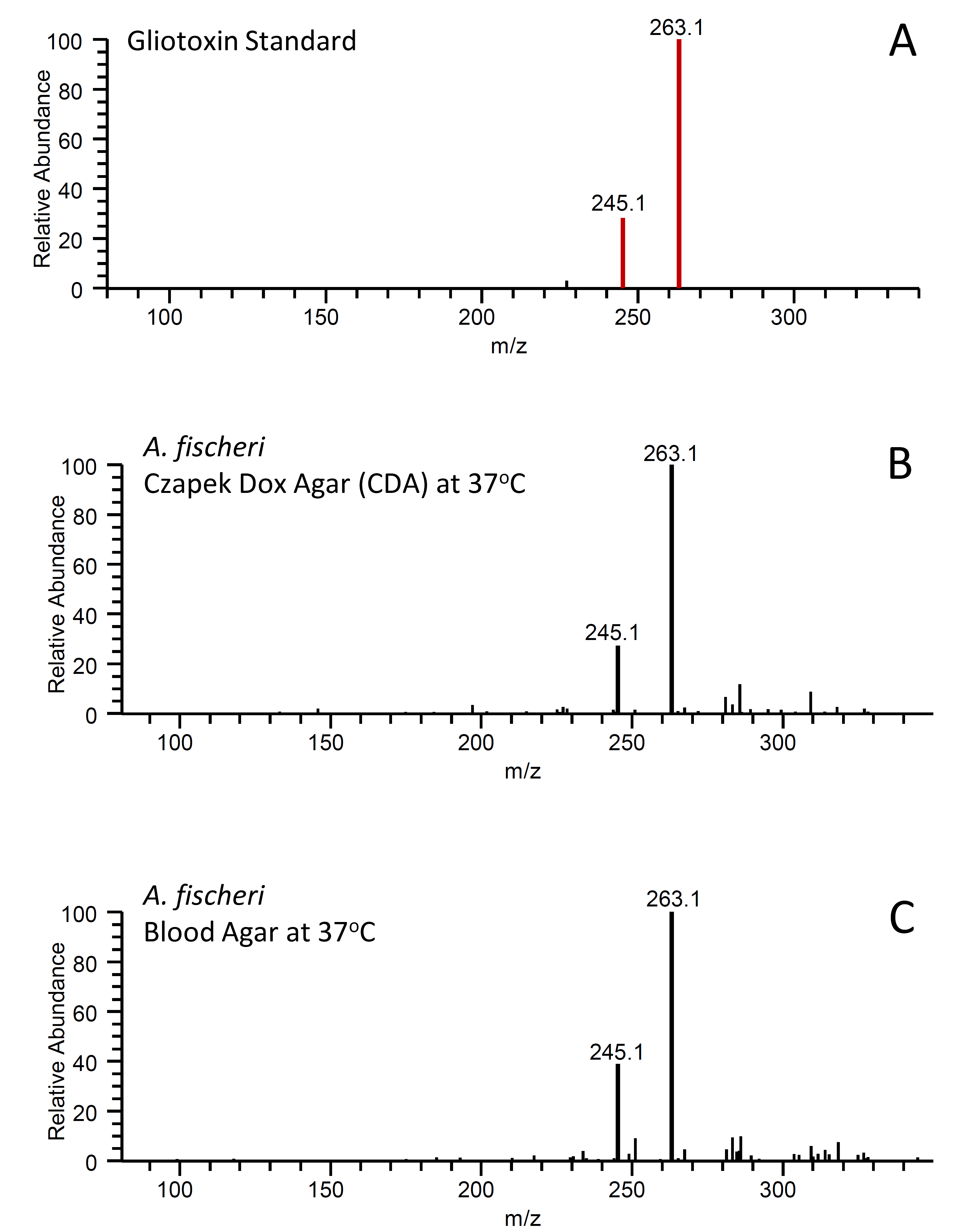

Supplement: FIG S5 [file mBio.03361-19-sf005.docx]
